# Field Examinations on the Application of Novel Biochar-Based Microbial Fertilizer on Degraded Soils and Growth Response of Flue-Cured Tobacco (*Nicotiana tabacum* L.)

**DOI:** 10.3390/plants13101328

**Published:** 2024-05-11

**Authors:** Xu Yang, Ke Zhang, Zhiming Qi, Hiba Shaghaleh, Chao Gao, Tingting Chang, Jie Zhang, Yousef Alhaj Hamoud

**Affiliations:** 1 College of Hydrology and Water Resources, Hohai University, Nanjing 210024, China; yangx@hhu.edu.cn (X.Y.); yousef-hamoud11@hhu.edu.cn (Y.A.H.); 2 The National Key Laboratory of Water Disaster Prevention, Hohai University, Nanjing 210024, China; 3 China Meteorological Administration Hydro-Meteorology Key Laboratory, Hohai University, Nanjing 210024, China; 4 Yangtze Institute for Conservation and Development, Hohai University, Nanjing 210024, China; 5 Key Laboratory of Water Big Data Technology of Ministry of Water Resources, Hohai University, Nanjing 210024, China; 6 Department of Bioresource Engineering, McGill University, Montreal, QC H9X 3V9, Canada; zhiming.qi@mcgill.ca; 7 College of Environment, Hohai University, Nanjing 210024, China; hiba-shaghaleh@njfu.edu.cn; 8 Institute of Geographical Sciences, Henan Academy of Sciences, Zhengzhou 450052, China; superagao@hhu.edu.cn; 9 College of Agricultural Science and Engineering, Hohai University, Nanjing 211100, China; changtt@hhu.edu.cn (T.C.); zhangjiejxd@hhu.edu.cn (J.Z.)

**Keywords:** biochar-based microbial fertilizer, photosynthetic rate, soil water availability, yield, *Nicotiana tabacum* L.

## Abstract

Southwestern China is receiving excessive chemical fertilizers to meet the challenges of continuous cropping. These practices are deteriorating the soil environment and affecting tobacco (*Nicotiana tabacum* L.) yield and quality adversely. A novel microbially enriched biochar-based fertilizer was synthesized using effective microorganisms, tobacco stalk biochar and basal fertilizer. A field-scale study was conducted to evaluate the yield response of tobacco grown on degraded soil amended with our novel biochar-based microbial fertilizer (BF). Four treatments of BF (0%, 1.5%, 2.5% and 5%) were applied in the contaminated field to grow tobacco. The application of BF_1.5_, BF_2.5_ and BF_5.0_ increased the available water contents by 9.47%, 1.18% and 2.19% compared to that with BF_0_ respectively. Maximum growth of tobacco in terms of plant height and leaf area was recorded for BF_1.5_ compared to BF_0_. BF_1.5_, BF_2.5_ and BF_5.0_ increased SPAD by 13.18–40.53%, net photosynthetic rate by 5.44–60.42%, stomatal conductance by 8.33–44.44%, instantaneous water use efficiency by 55.41–93.24% and intrinsic water use efficiency by 0.09–24.11%, while they decreased the intercellular CO_2_ concentration and transpiration rate by 3.85–6.84% and 0.29–47.18% relative to BF_0_, respectively (*p* < 0.05). The maximum increase in tobacco yield was recorded with BF_1.5_ (23.81%) compared to that with BF_0_. The present study concludes that the application of BF_1.5_ improves and restores the degraded soil by improving the hydraulic conductivity and by increasing the tobacco yield.

## 1. Introduction

Southwestern China is the largest concentrated and contiguous karst region in the world [1]; due to limitations of cultivated land area, economic benefits, and climatic restrictions, continuous cropping is commonly conducted in karst mountainous areas. Perennial continuous cropping constraints (often termed ‘continuous cropping obstacle’) have significantly damaged the soil environment in agroecosystems. Continuous cropping is one of the main factors restricting the sustainable development of agriculture around the world [2]. Meanwhile, to increase agricultural productivity, large amounts of chemical fertilizer have been applied to arable land over the last few decades [3]. However, excessive chemical fertilizer application does not always increase the crop yield but changes soil hydraulic parameters and inhibits crop growth, and photosynthesis is a major cause of crop yield and quality decline [4]. Flue-cured tobacco (*Nicotiana tabacum* L.) is China’s most important economic crop. China has the largest cultivated area of tobacco and yield in the world [5], and tobacco leaf production plays a vital role in China’s agriculture and national economy [2]. The two constraints of long-term continuous cropping and excessive chemical fertilizer application affect photosynthetic characteristics and influence the yield of flue-cured tobacco [6]. Therefore, it is urgent to formulate a promising alternative fertilizer for alleviating the two constraints and their adverse effects on the karst soil environment, thereby improving tobacco growth, photosynthetic characteristics and yield.

Biochar or biochar fertilizers are widely used in tobacco fields and have been proven to improve both the growth and yield of flue-cured tobacco [6]. For instance, the application of biochar improved the soil nutrient status and promoted fungal community diversity in tobacco-planting soils [5]. Biochar applied in soil increased pH, CEC, porosity and rhizosphere bacteria abundance, decreased bulk density and reduced the incidence and severity of tobacco bacterial wilt disease [7]. Similarly, this application decreased the total sugar content and increased the leaf K content, thereby significantly improving the leaf quality of flue-cured tobacco [8]. Notably, reports are available where biochar-based fertilizer is added to tobacco-planting soil and helps reduce nutrient leaching [9]. Similar findings were observed that biochar-based fertilizer has proven to enhance crop yields [10] and change the soil properties (pH, nutrients, organic matter, structure, etc.) [11]. Thus far, although many reviews touching on various issues related to biochar-based fertilizers have been published, most studies have focused on biochar as a microbial fertilizer carrier or chemical fertilizer carrier to prepare biochar-based fertilizers [12,13]. However, research on the formulation of fertilizers based on the combination of biochar, effective microorganisms and basal fertilizer is comparatively limited, and the degree of change in soil hydraulic parameters and enhancement remaining in the production of flue-cured tobacco is unclear. Biochar is widely recognized for its large specific surface area, porous microstructure and abundant large surface functional groups [14]. If we combine biochar with microbes [15], the large specific surface area and porous microstructure can provide a favorable habitat for microbes, which may be a new biochar-based microbial fertilizer solution to improve the karst soil environment and enhance crop growth affected by two constraints.

Therefore, a promising alternative fertilizer, formulated for alleviating the two constraints and their adverse effects on the karst soil environment, will provide a new improvement strategy for these problems (e.g., the deterioration of the karst soil environment and the decline in crop yield and quality), thereby improving tobacco growth, photosynthetic characteristics and yield. We hypothesized that the different ratios of biochar-based microbial fertilizer have different effects on the soil hydraulic parameters and the growth of flue-cured tobacco. Hence, we designed four levels of biochar-based microbial fertilizer to illuminate the influences. The purpose was to build a novel biochar-based microbial fertilizer that could replace chemical fertilizer through a formulation of biochar, effective microorganisms and basal fertilizer. The correlation between biochar-based microbial fertilizer and agronomic traits, SPAD values, photosynthetic characteristics, WUE and yield served as the theoretical basis used to develop this fertilizer.

The objectives of this study were (1) to clarify the effect of different levels of biochar-based microbial fertilizer on soil hydraulic properties, (2) to reveal the theoretical relationship between tobacco growth and soil hydraulic properties, and (3) to develop a novel biochar-based microbial fertilizer to reduce the impact of degraded soil caused by continuous cropping and excessive chemical fertilizer. This study is expected to provide new insights for strategies to improve soil hydraulic parameters and plant growth or mitigate the adverse effects of continuous cropping and excessive chemical fertilizer application on plants, as well as formulate novel biochar-based microbial fertilizers that are innocuous to the karst soil environment.

## 2. Materials and Methods

### 2.1. Experimental Site

The experiment was carried out from April to September 2018 at the Hohai University and Institute of Tobacco Science in Guizhou Province Experimental Station (26°45′ N, 103°59′ E), Guizhou, China. The altitude of the test base is 2212 m, which belongs to the subtropical monsoon humid climate zone. The experimental site has an organic matter content of 23.07 g kg^−1^, total nitrogen of 1.59 g kg^−1^, available nitrogen of 109.56 mg kg^−1^, available phosphorus of 20.20 mg kg^−1^, available potassium of 102.90 mg kg^−1^ and pH of 7.23. The soil is classified (based on FAO taxonomic classification) as Mollie Gleysols in the experimental site. The climatic variables of the flue-cured tobacco growing season from April to September were measured at the meteorological station near the test site, with monthly average precipitation of 117.78 mm, monthly average air temperature of 16.22 °C and monthly total sunshine hours of 125.98 h.

### 2.2. Biochar-Based Microbial Fertilizer Preparation

The biochar-based microbial fertilizer used in this study was produced by the formulation of biochar, effective microorganisms (EM) and basal fertilizer for flue-cured tobacco. The biochar was manufactured from tobacco stalks, and air-dried tobacco stalks were carbonized at a temperature of 500 °C under limited-oxygen conditions in the Muffle furnace. Biochar had a pH of 9.15, the conversion rate of flue-cured tobacco stems into biochar was about 43.7%, particle size distribution of 30.48% fine sand (0.075–2 mm) and 68.52% silt (0.075 mm). In this study, the biochar–EM bokashi–basal fertilizer mixed granules had a mass ratio of 10:0.75:40. Then, we added 1.5%, 2.5% and 5% of EM liquid to mixed granules according to the mass ratio, fermented in a closed container at 25 °C for 15 days, thus producing the 1.5%, 2.5% and 5% biochar-based microbial fertilizer (BF_1.5_, BF_2.5_ and BF_5.0_).

The EM had a yellow-brown liquid with a pH of 3.8 and emitted the smell of kvass or fermenting fruit juice (*photosynthetic bacteria*, *yeasts*, *actinomycetes* and *fermenting fungi*, etc.) (provided by EM SYSTEMS–Aiyile Environmental Biotechnology (Nanjing) Co., Ltd., Nanjing, China). EM bokashi is produced by fermenting EM liquid (diluted 50–100-times), molasses and rice husk at a mass ratio of 1:1:400, and adding distilled water at a mass ratio of 30% for 15 days. The basal fertilizer was provided by the Tobacco Institute of Science in Guizhou Province; basal fertilizer had an N of 9%, P_2_O_5_ of 13%, K_2_O of 22% and total nutrient content of 43.5%.

### 2.3. Experimental Design

The experiment was in a completely randomized design. Treatments consisted of four levels of biochar-based microbial fertilizer (BF) application. The biochar-based microbial fertilizer application rate, in this study, was approximately 750 kg hm^−2^, the suggested biochar-based microbial fertilizer application for tobacco by the tobacco industry and Chen et al. [16]. The field treatments were designed as follows: (1) CK (BF_0_); (2) BF_1.5_, 750 kg hm^−2^ of biochar-based microbial fertilizer; (3) BF_2.5_, 750 kg hm^−2^ of biochar-based microbial fertilizer; (4) BF_5.0_, 750 kg hm^−2^ of biochar-based microbial fertilizer. To ensure sufficient nutrient supply during the experiment, all treatments received a uniform application of 600 kg hm^−2^ N:P_2_O_5_:K_2_O = 9:13:22 as basal fertilizer. All the BF treatments supplemented P_2_O_5_ and K_2_O with calcium superphosphate and potassium sulphate, respectively.

The tobacco (*Nicotiana tabacum* L., cultivar ‘Yunyan 87’, and the seeds provided by the Guizhou Academy of Tobacco Science) seedlings were cultivated in polystyrene trays with the substrate in the float system for tobacco transplants. Before the experiment, the field had been planted with tobacco continuously for over ten years. Seedling transplanting was performed on 26 April 2018, with spacing between lines of 1.1 m and plant spacing of 0.55 m. When the tobacco plants grew to 23–25 leaves, they were topped (removal of flowers at the top of the plant), 2–3 useless basal leaves were removed and 20 effective leaves were reserved per plant. Tobacco production was managed following the local conventional practice, except for biochar-based microbial fertilizer use, and it was consistent across all treatments.

### 2.4. Soil Hydraulic Parameters

The soil water characteristic curve (SWCC) was determined by using pressure plates (Pressure Vessel 1500, Soil Moisture Equipment Corporation, Goleta, CA, USA), following the method of Yang et al. [17]. Suction was successively applied to establish ten matric potentials, namely, 0, 0.01, 0.02, 0.04, 0.06, 0.08, 0.1, 0.3, 0.6, 0.9, 1.2 and 1.5 MPa. Ultimately, all the soil samples were maintained at 105 °C until reaching a constant mass, weighed on three occasions and the mean was used to calculate the soil’s volumetric water content at each pressure level (saturation to wilting) [18]. These soil moisture contents and corresponding pressures were used to create an SWCC.

By plotting *θ* against soil matrix suction (*ψ_m_*), SWCC reflects the internal relations between the energy and quantity of soil water. The soil water characteristic curve was fitted using the Gardner model [18]:

(1) $$\theta =A\cdot {\psi_{m}}^{-B}$$

where *θ* is the soil water content (cm^3^ cm^−3^), *ψ_m_* is the soil matrix suction (MPa) and *A*, *B* are parameters that denote the shape of the SWCC.

Soil water characteristic parameters were derived from the SWCCs. These parameters included moisture at saturation (*θ_sat_* defined as the equilibrium volumetric soil water content *ψ_m_* at a matric potential of −0 bar), field capacity (*θ_fc_* defined as the equilibrium volumetric soil water content *ψ_m_* at a matric potential of −0.3 bar), capillary fracture (*θ_cp_*, is about 65% of *θ_fc_*), permanent wilting point (*θ_pw__p_*, volumetric soil water content *ψ_m_* at a matric potential of −15 bar) and hygroscopic coefficient (*θ_hyg_*, *θ_pw__p_* is the soil moisture at which the plant will be permanently wilted and *θ* has reduced to 1.5- to 2.0-times the soil’s *θ_hyg_*) [18]. The gravity water (*θ_gw_*) was calculated as the difference between the *θ_sat_* and *θ_fc_*, available water (*θ_aw_*) was calculated as the difference between the *θ_fc_* and *θ_pw__p_* and unavailable water (*θ_uaw_*) with water less than *θ_pw__p_* [18,19].

### 2.5. Agronomic Traits, SPAD Value, Photosynthetic Characteristics and Yield

Agronomic traits of flue-cured tobacco were as follows: each treatment used 15 tobacco plants to determine the plant height, stem girth, the largest leaf length and width of flue-cured tobacco (at the growth stage, i.e., on the days after transplanting (DAT) of 56 and maturity stage on 75 DAT). The flue-cured tobacco leaf area per plant calculation method is as follows [2]. When the tobacco leaves matured, they were placed in an incubator at 105 °C for 0.5 h and subsequently dried at 75 °C until reaching a constant weight; the dry matter accumulation could be obtained, which was then used to calculate the yield.

In the growth stage, photosynthetic characteristics were measured on the same leaves using the LI-6400 portable photosynthetic apparatus from around 8:30 to 11:30 a.m. The measured leaf was the most recently fully expanded. This experiment used the LI-6400 portable photosynthetic device manufactured by the American company LI-COR; the light intensity was controlled at around 800 µmol m^−2^ s^−1^. Characteristics measured included the net photosynthetic rate (*Pn*), stomatal conductance (*Gs*), intercellular CO_2_ concentration (*Ci*) and transpiration rate (*Tr*) [2]. Following Hoover et al. [20], the *Pn*/*Tr* ratio was taken as an estimate of instantaneous water use efficiency (*WUE_ins_*) and the ratio between *Pn*/*Gs*, which is known as intrinsic water use efficiency (*WUE_in_*). In the growth stage, the same leaves were chosen to measure the SPAD value with a portable chlorophyll meter (SPAD-502, Minolta, Tokyo, Japan) according to the method of Nakanishi et al. [21].

### 2.6. Statistical Analysis

Statistical analysis for data collected from the completely randomized design experiment was conducted by using SPSS 17.0 (SPSS Inc., Chicago, IL, USA). The significance of differences in measured parameters among treatments was evaluated by the one-way ANOVA followed by Duncan’s Multiple Range Test at *p* < 0.05. Data were presented as means ± S.E. (standard error). Pearson’s correlation coefficients were assumed as statistically significant at *p* < 0.05. The relationship between soil hydraulic properties and the growth performance of flue-cured tobacco was analyzed by using the redundancy analysis (RDA) from Canoco 5.0 (version 5.0, Wageningen, The Netherlands).

## 3. Results

### 3.1. Changes in Soil Water Characteristic Curve

**Figure 1:**
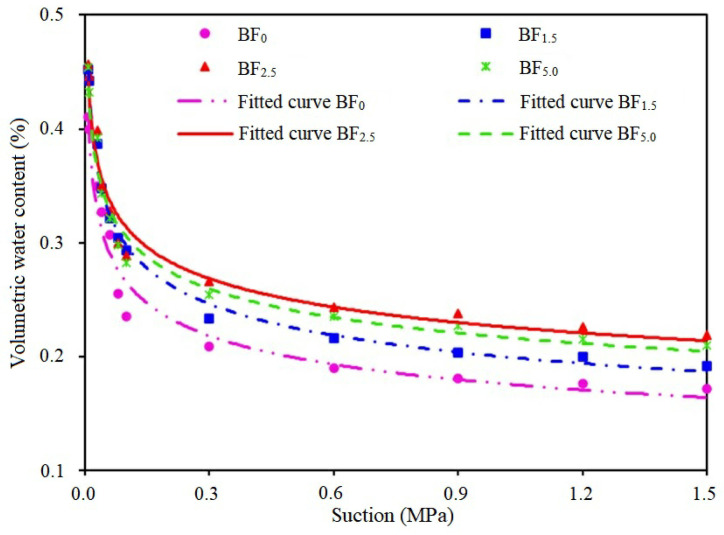
Soil water characteristic curve within the suction range of 0–1.5 (MPa).

The changes in the soil water characteristic curve (SWCC) under different biochar-based microbial fertilizer (BF) treatments are shown in Figure 1, and the parameters (A, B) of each treatment fitted by the Gardner model are provided in Table 1. As can be seen in Figure 1, the differences between the SWCC of treated soil appeared not only within a high matric potential range but also in a low range below 1.5 MPa. Specifically, the SWCCs of BF_1.5_, BF_2.5_ and BF_5.0_ were higher than BF_0_, which indicated that the biochar-based microbial fertilizer application improved SWCCs when compared with the BF_0_ control. With an increase in biochar-based microbial fertilizer application, the soil water holding capacity of BF_5.0_ was decreased, which was lower than BF_2.5_, as shown in Figure 1. Nevertheless, this change was still greater than that found for the water holding capacity with the BF_0_ treatment. Moreover, the Gardner model parameter (A) in BF_1.5_, BF_2.5_ and BF_5.0_ was increased by 13.63%, 28.45% and 23.23%, respectively, in comparison with BF_0_ (Table 1). From the SWCC and the parameters, the soil water holding capacity under the final matrix suction could be ordered as BF_2.5_ > BF_5.0_ > BF_1.5_ > BF_0_, indicating that biochar-based microbial fertilizer application increased the soil water holding capacity. This is because the biochar-based microbial fertilizer has a large specific surface area and functional groups on the surface and is rich in a variety of effective microorganisms, which has caused changes in the karst soil environment.

The SWCCs were fitted using the Gardner model, and the correlation coefficients of the soil water characteristic curves were R^2^ > 0.97 (Table 1), indicating that the data that fitted the soil water characteristic curves were closely correlated to the measured data.

### 3.2. Changes in Soil Water Characteristic Parameters and Soil Water Availability

Soil water characteristic parameters were influenced by the application of biochar-based microbial fertilizer (Table 2). Compared with the BF_0_ control, the application of biochar-based microbial fertilizer led to alterations in the soil hydraulic characteristic parameters, with varying effects on different parameters observed across different application rates. Specifically, applied BF_1.5_ increased the *θ_sat_*, *θ_fc_*, *θ_cf_*, *θ_pwp_* and *θ_hyg_* by 0.0416, 0.0369, 0.0240, 0.0200 and 0.0125 cm^3^ cm^−3^, respectively, in comparison with BF_0_ (Table 2). In contrast, applied BF_2.5_ was significantly higher than BF_1.5_, which increased the *θ_sat_*, *θ_fc_*, *θ_cf_*, *θ_pwp_* and *θ_hyg_* by 0.0464, 0.0489, 0.0262, 0.0468 and 0.0293 cm^3^ cm^−3^, respectively, in comparison with BF_0_ (Table 2). Similar trends were observed in BF_5.0_, which increased the *θ_sat_*, *θ_fc_*, *θ_cf_*, *θ_pwp_* and *θ_hyg_* by 0.0438, 0.0423, 0.0275, 0.0384 and 0.0241 cm^3^ cm^−3^, respectively, as compared to BF_0_ (Table 2).

The different biochar-based microbial fertilizer treatments also exerted different effects on soil water availability, as shown in Table 2. The application of biochar-based microbial fertilizer generally increased gravity water (except BF_2.5_), available water and unavailable water. Although the gravity water of each treatment is not proportional to the amount of biochar-based microbial fertilizer applied, as shown in Table 2, this did not affect the finding that available water was strongly affected by the ratio of biochar-based microbial fertilizer applied. The soil available water is a water resource that is available to plants and can well reflect the soil water holding performance. Notably, available water for the application of BF_1.5_, BF_2.5_ and BF_5.0_ was increased by 9.47%, 1.18% and 2.19% compared to BF_0_ (Table 2). All of those findings indicate that the application of biochar-based microbial fertilizer could increase soil water availability. Consequently, we thus concluded that this finding also shows that the application of biochar-based microbial fertilizer can improve the soil hydraulic environment to alleviate the adverse effects of continuous cropping/excessive chemical fertilizer application in the karst ecosystem.

### 3.3. Changes in Plant Height, Stem Girth and Leaf Area

**Figure 2:**
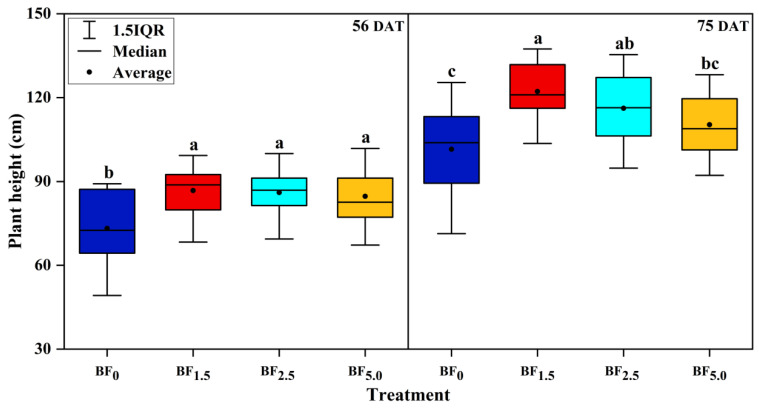

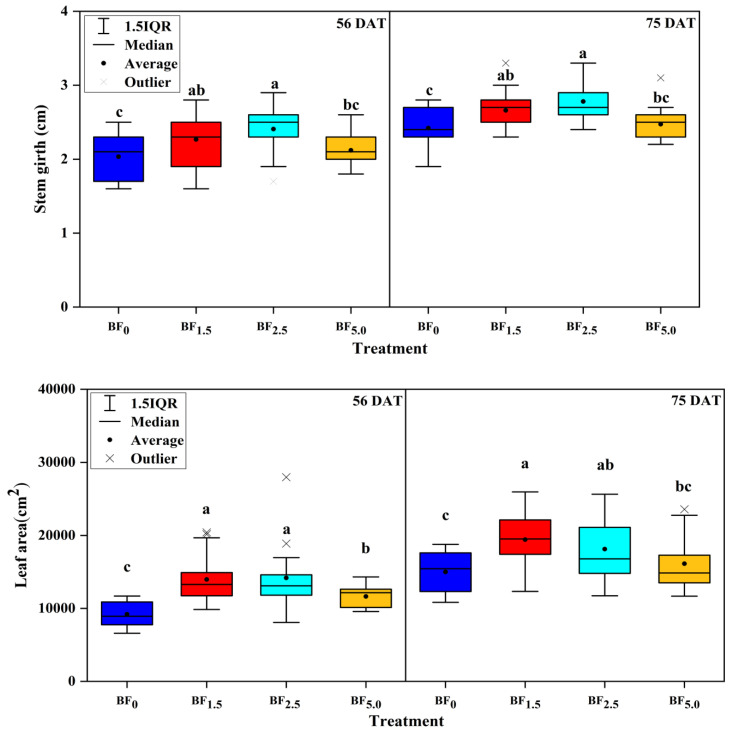
Effects of biochar-based microbial fertilizer on plant height, stem girth, and leaf area of flue-cured tobacco. Note: Columns with the same letters are not significantly different by Duncan’s Multiple Range Test at *p* < 0.05.

The changes in plant height, stem girth and leaf area along with the days after transplanting (DAT) under different biochar-based microbial fertilizer (BF) treatments are shown in Figure 2. ANOVA analysis revealed that application of 1.5% biochar-based microbial fertilizer significantly increased plant height and stem girth, respectively, by 18.58% and 11.48% at the growth stage, i.e., on the 56 DAT, and at the maturity stage (75 DAT), increased by 20.33% and 9.92%, compared with those of control (BF_0_), respectively (*p* < 0.05) (Figure 2). Similar findings were observed at a higher ratio of biochar-based microbial fertilizer (2.5%), increased plant height and stem girth, respectively, by 17.59% and 18.36% (56 DAT), 14.42% and 14.88% (75 DAT) (*p* < 0.05). In contrast, the high ratio of biochar-based microbial fertilizer (5%) significantly increased the plant height by 15.74% (56 DAT) (*p* < 0.05), or no significant effects on plant height (75 DAT) and stem girth (both 56 and 75 DAT) (*p* > 0.05) (Figure 2). The results indicated that the application of BF_1.5_ was recognized as the best BF treatment in our study. This suggests that the low BF ratios (BF_1.5_ and BF_2.5_) positively influenced the plant height and stem girth of flue-cured tobacco. In comparison, high ratios (BF_5.0_) inhibited the plant height and stem girth compared with low ratios that may correspond to the effective microorganisms (5%) in biochar-based microbial fertilizer-induced acidity.

It is well known that the leaf area directly affects the yield of flue-cured tobacco and is an important factor in the value of flue-cured tobacco. Applications of 1.5%, 2.5% and 5% biochar-based microbial fertilizer significantly increased the leaf area by 51.78%, 54.03% and 26.41%, as compared with that of the control (BF_0_) (*p* < 0.05), respectively, at the growth stage, i.e., on the 56 DAT (Figure 2). In the maturity stage (75 DAT), the leaf area of flue-cured tobacco was still somewhat increased and tended to be stable. Application of 1.5%, 2.5% and 5% biochar-based microbial fertilizer increased the leaf area by 29.52%, 20.81% and 7.50%, as compared to with that of the control (*p* < 0.05), although a 7.50% increase was not statistically significant (*p* > 0.05).

### 3.4. Changes in SPAD Values

**Figure 3:**
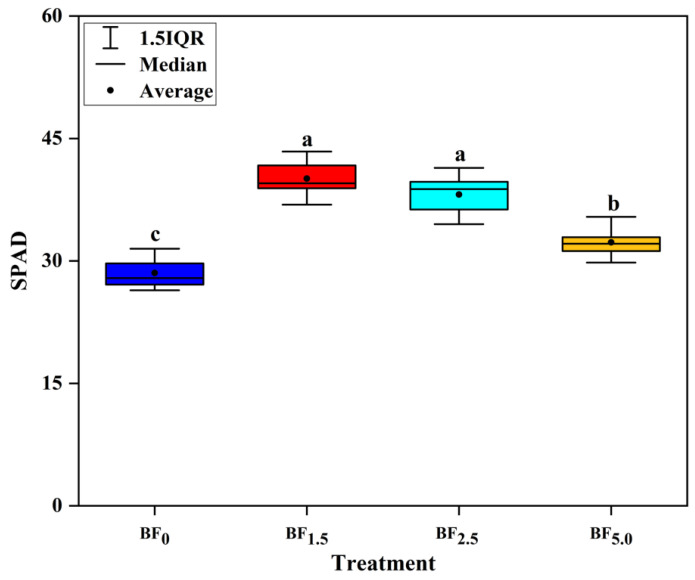
Effects of biochar-based microbial fertilizer on SPAD values of flue-cured tobacco. Note: Columns with the same letters are not significantly different by Duncan’s Multiple Range Test at *p* < 0.05.

Biochar-based microbial fertilizer (BF) had a significant effect on leaf SPAD values (Figure 3). The SPAD values increased from 28.52 to 40.08 and 38.14 as the biochar-based microbial fertilizer level increased from BF_0_ to BF_1.5_ and BF_2.5_, while it decreased from 38.14 to 32.28 as the biochar-based microbial fertilizer level increased from BF_2.5_ to BF_5.0_. Application of BF_1.5_, BF_2.5_ and BF_5.0_ significantly increased the SPAD values by 40.53%, 33.73% and 13.18% relative to the control (BF_0_), respectively (*p* < 0.05). However, it was observed that at high ratios of biochar-based microbial fertilizer, the increased acidity decreased the positive effect of biochar-based microbial fertilizer. This phenomenon is attributed to the high ratios of biochar-based microbial fertilizer, compounded with a high ratio of effective microorganisms. Consequently, applying high ratios of biochar-based microbial fertilizer inhibited the SPAD values of flue-cured tobacco.

### 3.5. Changes in Photosynthetic Characteristics

Compared to the control (BF_0_), the application of biochar-based microbial fertilizer treatments resulted in a higher leaf net photosynthetic rate (*Pn*), ranging from 10.47 to 15.93 μmol m^−2^ s^−1^ (Table 3). The maximum *Pn* value was in BF_1.5_-treated plants (Table 3), while the BF_2.5_ and BF_5.0_ were lower than BF_1.5_, indicating that the higher ratios of biochar-based microbial fertilizer inhibited the *Pn*. The *Gs* and *Pn* exhibited a similar tendency. The maximum *Gs* were recorded under the BF_1.5_ treatment (0.52 mol m^−2^ s^−1^) and the minimum under BF_0_ (0.36 mol m^−2^ s^−1^), while the value was not significantly different from the control (*p* > 0.05). The *Ci* was decreased with the ratios of biochar-based microbial fertilizer application. Increasing the ratio of biochar-based microbial fertilizer from BF_0_ to BF_1.5_ resulted in a decrease in *Ci* from 390 to 375 μmol mol^−1^, while increasing the ratio of biochar-based microbial fertilizer from BF_1.5_ to BF_2.5_ and BF_5.0_ resulted in a decrease in *Ci* from 375 to 367.67 and 363.33 μmol mol^−1^. Compared to BF_0_, the values of *Tr* in BF_1.5_ and BF_2.5_ increased by 1.13 and 0.2 mmol m^−2^ s^−1^ (*p* > 0.05), while decreased 2.11 mmol m^−2^ s^−1^ in BF_5.0_ (*p* < 0.05), the *Tr* decreased with the application of biochar-based microbial fertilizer, indicating that the application of biochar-based microbial fertilizer inhibited the *Tr*, while no significant differences were found in BF_1.5_ and BF_2.5_ (*p* > 0.05). The leaf instantaneous water use efficiency (*WUE_ins_*) varied from 1.48 (BF_0_) to 2.86 (BF_5.0_) μmol mmol^−1^, and the leaf intrinsic water use efficiency (*WUE_in_*) varied from 30.28 (BF_0_) to 37.58 (BF_2.5_) μmol mol^−1^. Higher *WUE_ins_* and *WUE_in_* were observed in BF_5.0_ and BF_2.5_. Specifically, the *WUE_ins_* in BF_1.5_, BF_2.5_ and BF_5.0_ was 55.41%, 69.59% and 93.24% higher than BF_0_, while the *WUE_in_* in BF_1.5_, BF_2.5_ and BF_5.0_ was 19.91%, 24.11% and 0.09% higher compared to BF_0_. This indicates that the quantitative relations of gas assimilation ability and tobacco growth with water use were high in higher ratios of BF, and similar in *WUE_in_*; the CO_2_ assimilation capability per unit of *Gs* was high in higher BF ratios, while a decrease was observed in BF_5.0_.

### 3.6. Changes in Yield

**Figure 4:**
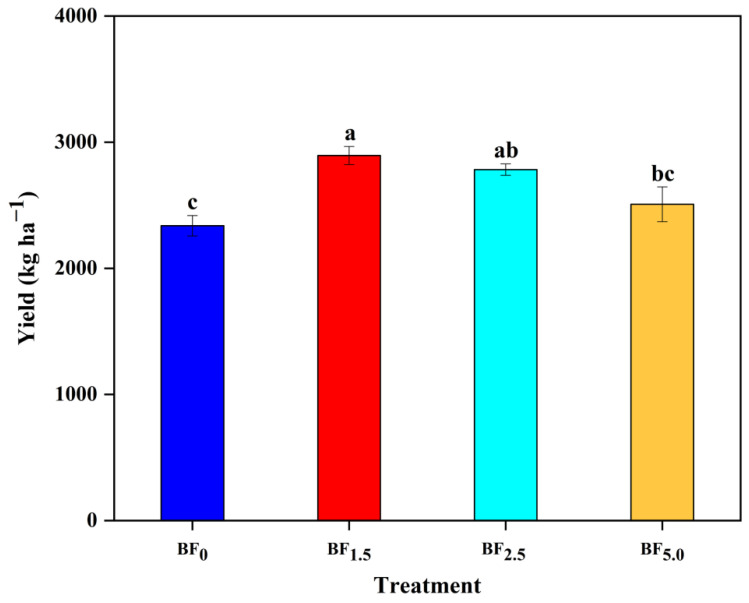
Effects of biochar-based microbial fertilizer on yield of flue-cured tobacco. Note: Columns with the same letters are not significantly different by Duncan’s Multiple Range Test at *p* < 0.05.

The application of 1.5% and 2.5% biochar-based microbial fertilizer significantly increased the tobacco yield by 23.81 and 19.03% relative to control (BF_0_), respectively (Figure 4). In contrast, high ratios of biochar-based microbial fertilizer (for BF_5.0_) had no significant effect or slightly increased the tobacco yield by 7.28% (*p* > 0.05). The lack of positive effects of high biochar-based microbial fertilizer ratios may correspond to the effective microorganisms (5%) in biochar-based microbial fertilizer-induced acidity. Low soil pH resulting from high biochar-based microbial fertilizer ratios can disturb the distribution and activity of beneficial soil microflora, impacting nutrient availability and ultimately affecting flue-cured tobacco yield. Interestingly, our data demonstrated that the increased tobacco yield in response to low-ratio effective microorganisms of biochar-based microbial fertilizer might improve the nutrient status by improving soil conditions. In other words, at high ratios of biochar-based microbial fertilizer, the flue-cured tobacco response will result from the interactive effect of some direct (the biochar supplied nutrients) or indirect (the high ratios effective microorganisms in biochar-based microbial fertilizer induced acidity, etc.) factors.

### 3.7. Pearson Correlations between Biochar-Based Microbial Fertilizer and Agronomic Traits, Photosynthetic Characteristics and Yield of Flue-Cured Tobacco

Table 4 shows the relationships between biochar-based microbial fertilizer and agronomic traits, SPAD values, photosynthetic characteristics, WUE and yield in flue-cured tobacco. Generally, the biochar-based microbial fertilizer was positively correlated with the agronomic traits, SPAD, photosynthetic characteristics, WUE and yield of flue-cured tobacco. However, the biochar-based microbial fertilizer was positively correlated with plant height, stem girth, leaf area, SPAD, *WUE_ins_* and yield, while negatively correlated with *Pn*, *Gs*, *Ci*, *WUE_in_* and significantly negatively correlated with *Tr* (r = 0.950 *, *p* = 0.050), suggesting that high ratios of biochar-based microbial fertilizer-induced acidity were the main limiting factor affecting agronomic traits, SPAD, photosynthetic characteristics, leaf WUE and yield in this study. The impact of low soil pH on the distribution and activity of beneficial soil microflora, as well as nutrient availability, likely played a role in these correlations. Moreover, plant height, stem girth, leaf area, SPAD, *Pn*, *Gs*, *WUE_ins_*, *WUE_in_* and yield were negatively correlated with *Ci* while positively correlated with other traits. This indicated that the *Ci* inhibited the leaf area, leaf WUE and yield. In addition, correlation analysis also demonstrated that the yield was negatively correlated with *Ci* while positively correlated with other traits. Specifically, the yield was significantly correlated with leaf area (r = 0.995 **, *p* = 0.005) and SPAD values (r = 0.999 **, *p* = 0.001) and significantly correlated with plant height (r = 0.984 *, *p* = 0.0168) and *Pn* (r = 0.977 *, *p* = 0.023). This indicates that leaf area, SPAD, plant height and *Pn* also affected the yield of flue-cured tobacco, especially leaf area and SPAD, as in our study. Therefore, increases in leaf area, SPAD, plant height and *Pn* are essential for achieving a high and stable yield of flue-cured tobacco.

### 3.8. Interpretations of Soil Hydraulic Properties and Agronomic Traits, Photosynthetic Characteristics and Yield by RDA Analysis

**Figure 5:**
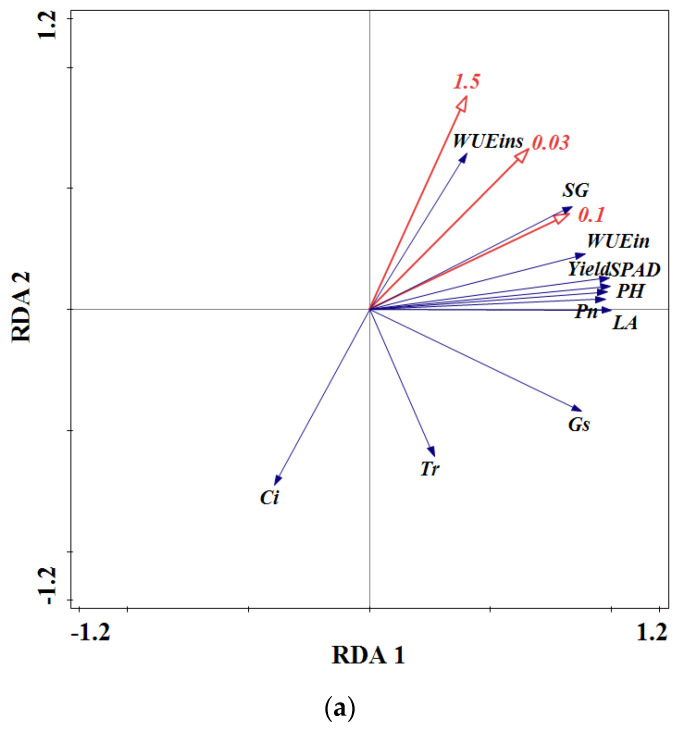

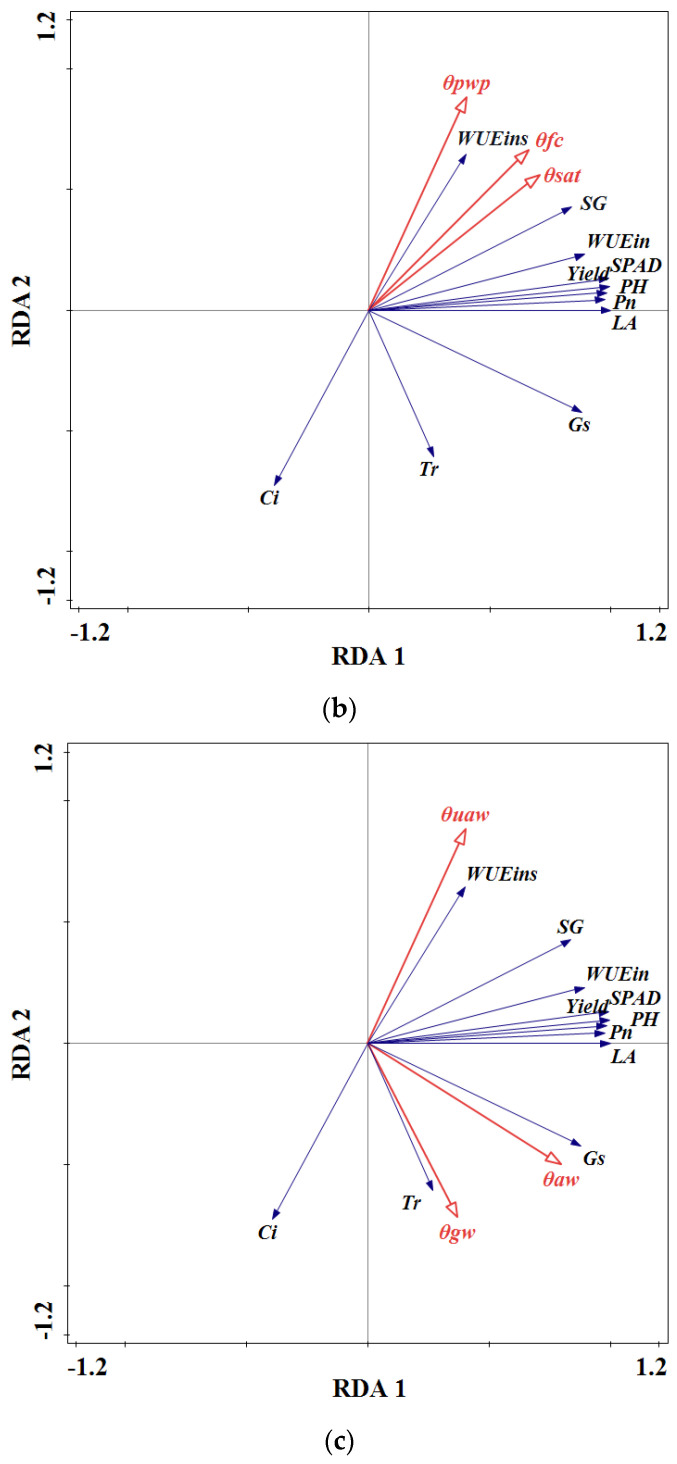
Redundancy analysis (RDA) demonstrating the relationships between soil water holding capacity (**a**), soil water characteristic parameters (**b**), soil water availability (**c**) and agronomic traits, photosynthetic characteristics and yield of flue-cured tobacco. Note: The numbers 0.03, 0.1 and 1.5 represent the soil water holding capacity of each soil suctions (Mpa); PH, SG, LA, *SPAD*, *Pn*, *Gs*, *Ci*, *Tr*, *WUE*_ins_ and *WUE_in_* are plant height, stem girth, leaf area, chlorophyll content, net photosynthetic rate, stomatal conductance, intercellular CO_2_ concentration, transpiration rate, leaf instantaneous water use efficiency and leaf intrinsic water use efficiency, respectively; *θ_sat_* represents saturated moisture, *θ_fc_* represents field capacity, *θ_cf_* represents capillary fracture moisture, *θ_pwp_* represents permanent wilting point, *θ_hyg_* represents hygroscopic coefficient, *θ_gw_* represents gravity water, *θ_aw_* represents available water, *θ_uaw_* represents unavailable water.

The soil hydraulic properties’ environment could influence the agronomic traits, photosynthetic characteristics and yield of flue-cured tobacco. The correlation between the soil hydraulic environment and agronomic traits, photosynthetic characteristics and yield of flue-cured tobacco was evaluated by RDA (Figure 5). In this study, soil environmental factors have significant effects on tobacco growth, namely soil water holding capacity (Figure 5a), soil water characteristic parameters (Figure 5b) and soil water availability (Figure 5c). RDA indicated that soil water holding capacity affected the agronomic traits, photosynthetic characteristics and yield of flue-cured tobacco (Figure 5a). Three suctions were selected from all the suctions as representatives to explain the soil water holding capacity. The 0.1 MPa was 68.4% (*p* < 0.01), followed by 0.03 MPa (43.5%) (*p* < 0.05) and 1.5 MPa (16.2%) (*p* > 0.05). In the ordination diagrams, soil water holding capacity exhibited a positive correlation with plant height, stem girth, leaf area, SPAD, *Pn*, Gs, *WUE_ins_*, *WUE_in_* and yield (Figure 5a). The application of biochar-based microbial fertilizer is an important factor in improving the soil water holding capacity. Increases in agronomic traits, photosynthetic characteristics and yield of flue-cured tobacco depend on the soil water holding capacity, and the change in soil water holding capacity will affect the growth of flue-cured tobacco. Therefore, biochar-based microbial fertilizer alters the soil water holding capacity and alleviates the adverse effects of continuous cropping/excessive chemical fertilizer application on the soil hydraulic environment.

RDA was conducted to further quantify the effects of soil water characteristic parameters on agronomic traits, photosynthetic characteristics and yield of flue-cured tobacco (Figure 5b). RDA results revealed that various soil water characteristic parameters exerted similar effects on agronomic traits, photosynthetic characteristics and yield of flue-cured tobacco (Figure 5b). In the ordination diagrams, parameters, such as *θ_sat_*, *θ_fc_* and *θ_pwp_*, exhibited a strong positive correlation with plant height, stem girth, leaf area, SPAD, *Pn*, *WUE_ins_*, *WUE_in_* and yield while a negative correlation with *Tr* and *Ci*. Moreover, RDA was conducted to determine the effects of the soil water availability on agronomic traits, photosynthetic characteristics and yield of flue-cured tobacco (Figure 5c). As can be seen in Figure 5c, the RDA illustrates that the plant height, stem girth, leaf area, SPAD, *Pn*, *Gs*, *Tr*, *WUE_in_* and yield increased with an increase in *θ_aw_*, whereas *Ci* and *WUE_ins_* displayed negative correlation with *θ_aw_*. A similar trend was detected in *θ_gw_*, which displayed positive correlations with plant height, leaf area, SPAD values, *Pn*, *Gs*, *Tr*, *Ci*, *WUE_in_* and yield, and showed negative correlations with stem girth and *WUE_ins_*. By contrast, *θ_uaw_* exhibited negative correlations with *Gs*, *Tr* and *Ci*.

## 4. Discussion

### 4.1. Biochar-Based Microbial Fertilizer Improved the Soil Hydraulic Properties

The soil water holding capacity increased after biochar-based microbial fertilizer application; this is mainly because biochar-based microbial fertilizer improves soil water retention by modifying the shape and sizes of soil pores, increasing total porosity and increasing aggregate formation and stability [22,23]. This is consistent with the findings of Kang et al. [24], who showed that biochar-based soil amendments improved soil water retention by 48.2–128.9% compared to the control. Interestingly, the soil water holding capacity of BF_5.0_ treatment was decreased compared with BF_2.5_ treatment, probably due to the greater soil acidity caused by the higher concentration of biochar-based microbial fertilizer with high ratios of effective microorganisms. In addition, the improvement in soil nutrient contents and bacterial community diversity induced by the biochar-based microbial fertilizer application could elevate the soil water characteristic parameters (*θ_sat_*, *θ_fc_*, *θ_cf_*, *θ_pwp_* and *θ_hyg_*) [15]. This was in accordance with previous studies that the application of fertilizer and biochar improved the soil water properties, such as field capacity (+20.4%) and permanent wilting point (+16.7%) [25]. Furthermore, it was observed that biochar-based microbial fertilizer application could enhance the soil water availability, benefiting from the small size of the particles inside; the pores have high capillary action or suction, and the tight attachment of water in the pores inside the particles helps to improve the soil water availability [26]. This finding was in line with the result of Edeh and Mašek [22], who also reported that biochar improved all investigated soil water properties, such as available water content (+28.5%). Similar studies were also found in Kang et al. [24], and soil water retention and plant water availability were improved for plant growth by biochar-based fertilizer.

### 4.2. Biochar-Based Microbial Fertilizer Improved the Agronomic Traits of Flue-Cured Tobacco

The plant height, stem girth and leaf area of flue-cured tobacco are crucial for tobacco leaf harvesting [2]. In this study, although the effect of biochar-based microbial fertilizer (i.e., 1.5%, 2.5% and 5%) on flue-cured tobacco’s plant height, stem girth and leaf area was not consistent across those three treatments, all biochar-based microbial fertilizer applications played an improved role. Consistently, previous studies also showed that the application of EM biochar-based fertilizer (100, 300, 600 g pot^−1^) can increase the leaf by 7.93–65.51% compared with the CK (0 g pot^−1^) [27]. Other studies showed that the application of biochar-based fertilizers increased in stem height compared to the control (0 t ha^−1^), and biochar-based fertilizer (1.5 t ha^−1^) caused a significant increase in leaf area (35.1%) compared to the control (*p* < 0.05) [28]. However, it is important to note that the results obtained from other crops may not necessarily be applicable to flue-cured tobacco cultivation. In previous studies, the increased biomass of biochar was generally attributed to ease root penetration and development via decreasing the soil bulk density and increasing the soil porosity, improving the soil microbial environment, increasing soil water holding capacity and soil water availability and, subsequently, increasing plant height, stem girth and leaf area [29].

Interestingly, the leaf area in low-ratio biochar-based fertilizer was higher than that at a high ratio due to the increased soil acidity caused by high rates of fertilizer, and the acidity of soil affects the distribution and activity of soil beneficial microflora as well as the availability of nutrients [30,31]. Moreover, it should be noted that excessively high levels of biochar-based fertilizer application have a positive effect on leaf area relative to control, while they inhibited the leaf area of flue-cured tobacco compared with low levels [27,32]. Therefore, the results offer prospects to increase the plant height, stem girth and leaf area for flue-cured tobacco farming.

### 4.3. Biochar-Based Microbial Fertilizer Enhanced the SPAD Value of Flue-Cured Tobacco

Several studies demonstrated that biochar-based fertilizers could elevate the SPAD value of flue-cured tobacco [30,33,34], which is consistent with our study. As in this study, the application of biochar-based microbial fertilizer exhibited positive effects on the SPAD values of flue-cured tobacco [30]. However, the increase in the high ratio has no more significance than that at the low ratio. Similarly, Zhao et al. [34] indicated that the tobacco stem-derived biochar increased the content of chlorophyll in *B. striata*. As a result of the positive effect of biochar-based fertilizers on soil health, they have also been shown to enhance plant performances compared to unenriched biochars or conventional fertilizers [33]. Biochar-based fertilizer can also improve the plant physiological properties. Ndoung et al. [33] reported an increase in the SPAD index of flue-cured tobacco (*Nicotiana tabacum* L.) when soils were treated with a biochar-based fertilizer.

In addition, the SPAD value increased with increasing fertilization, which may be attributed to the promotion of nitrogen absorption in the base fertilizer by flue-cured tobacco after biochar-based fertilizer application [29]. This result is emphasized by Ren et al. [35], who reported that the SPAD value of the treatment with biochar-based fertilizer under drip irrigation is slightly higher than that of the control. Our data showed that the application of biochar-based microbial fertilizer to the soil effectively supplied N to flue-cured tobacco. A higher leaf SPAD value was recorded in the biochar-based microbial fertilizer treatments because of the higher nitrogen content. The results were in agreement with the findings of Ren et al. [35], who reported that biochar-based fertilizer significantly affected the SPAD value. Similarly, Zhang et al. [31] also demonstrated a positive effect of increased nutrient supply on the leaf SPAD content, most obvious when N was applied.

### 4.4. Biochar-Based Microbial Fertilizer Indirectly Improved the Photosynthetic Characteristics of Flue-Cured Tobacco

Photosynthetic characteristics are the basis of plant production and the major factor in determining crop yield composition [35]. Our data demonstrated higher leaf *Pn* and *Gs* in the biochar-based microbial fertilizer treatment than in the control. Similar findings were observed in Ndoung et al. [33], who reported a biochar-based fertilizer increase in the net photosynthetic rate (*Pn*), stomatal conductance (*Gs*), intercellular CO_2_ concentration (*Ci*) and transpiration rate (*Tr*) of tobacco. This increase in the physiological metabolism may be attributed to the water retention capacity of biochar-based fertilizer. This indicated that the leaf *Pn* and *Gs* are sensitive to the biochar-based microbial fertilizer, and a higher ratio of biochar-based microbial fertilizer in the soil could significantly reduce the leaf assimilation rate, consequently impacting the growth and yield. However, we observed the leaf *Ci* decreased with the application of biochar-based microbial fertilizer. These findings were confirmed by Ren et al. [36], who indicated that biochar treatment increased the *Pn* value of tobacco by 5.09–11.48% and increased the *Gs* by 9.26–11.44%, while it was decreased in *Ci* by 0.92–3.83%. Noticeably, our results indicated that the *Tr* decreased with the application of biochar-based microbial fertilizer, while there was an increasing trend in *WUE_ins_* and *WUE_in_*. These trends are beneficial for maintaining the internal water balance and leaf turgidity in tobacco. These results are consistent with a previous study that showed improved WUE at the leaf level by increasing *Gs* and *Pn* while decreasing *Tr*, reducing plant water consumption and increasing the yield [37]. The increase in *Pn* with biochar-based microbial fertilizer application was associated with an increase in *Gs* and a decrease in *Ci*, and this may be due to the fact that the biochar-based fertilizer increased the diffusion of CO_2_ from the atmosphere to the intercellular cavities by increasing the size of the stomatal apertures [31]. Reports are available where biochar-treated plants reduce the loss of water by stomatal closure and low *Tr*, helping to increase the nutrient retention and supplying capacity of the soil and, thus, support photosynthetic performance [38]. Interestingly, our results observed that the *Ci* was negatively correlated with all traits of our analysis except *Tr*. This is because the plant anatomical and/or physiological parameters might have been changed due to biochar-based microbial fertilizer application, thus reducing the *Ci*. Contrary to this, this finding was contrasted against the results of Saha et al. [38], who reported that the *Ci* was higher in a combination of biochar (full or half dose of biochar) and fertilizer (full recommended dose of fertilizer), as compared to control.

In addition, our results showed that both *WUE_ins_* and *WUE_in_* increased with increasing biochar-based microbial fertilizer ratios, as *Pn* increased or *Tr* decreased with increasing biochar-based microbial fertilizer ratios. The results were in agreement with those of Chen et al. [39], who stated that an appropriate fertilizer supply is recommended to improve the photosynthetic efficiency and enhance *WUE_ins_*. In contrast, similar to the results of the present study, Li et al. [40] demonstrated that fertilizer’s main effects resulted in greater *WUE_in_*.

### 4.5. Biochar-Based Microbial Fertilizer Increased the Yield of Flue-Cured Tobacco

The primary objective of flue-cured tobacco cultivation is to harvest high-quality leaves; high yields and quality of tobacco leaves have important economic value [2]. In this study, the effect of biochar-based microbial fertilizer (i.e., 1.5%, 2.5%, 5%) was an increased leaf yield at all fertilizer levels, by 23.81%, 19.03% and 7.28%, respectively. These findings are useful for tobacco farmers’ economic income. Consistently, previous studies also indicated that the application of EM biochar-based fertilizer increased the yield of flue-cured tobacco by 7.93–65.51% compared with CK (g pot^−1^) [27]. Similar findings were observed in Zhang et al. [29], who reported that biochar-based granular fertilizer increased the leaf yield of flue-cured tobacco by 14–39% in our adjacent research area. In previous studies, the yield increase using biochar-based fertilizer has been frequently attributed to the ease of root penetration and development via decreasing the soil bulk density and increasing the soil water holding capacity and soil water availability, with a subsequent increase in nutrient uptake [29]. Another effect of biochar-based fertilizer is their ability to improve tobacco growth at different stages, such as seedling, flowering and harvest [33]. Such behavior was attributed to the fact that the biochar-based fertilizer caused a slow nutrient release, more balanced nutrient fluxes and reduced nutrient losses [33].

It was interesting to note that the yield of flue-cured tobacco in low-ratio biochar-based microbial fertilizer was higher than that at high ratios. By contrast, the yield with the lower ratio (1.5%) was significantly higher than the higher ratio (5%), while the higher ratio (5%) was not significantly higher than CK (0%). These effects, i.e., the increase in yield at all levels of biochar-based microbial fertilizer application, indicate that the appropriate biochar-based microbial fertilizer is suitable for the growth of the leaves, but a high ratio is not suitable. Therefore, the results offer prospects to increase the yield for flue-cured tobacco farming. The results could be attributed to the slower release characteristics of biochar-based fertilizer synchronized with better N uptake by tobacco plants, which contributed to providing optimal nutrition for the growth of flue-cured tobacco leaves [29].

Our results demonstrated that the yield was negatively correlated with *Ci* while positively correlated with other traits. Similar studies by De Souza et al. [41] have revealed increased photosynthetic efficiency and biomass in tobacco in fields by accelerating recovery from photoprotection. Moreover, they also indicated an improved yield by up to 33% by increasing the photosynthetic efficiency [41]. Similarly, Wang et al. [42] illustrated that the application of biochar produced the highest yield by enhancing the leaf photosynthetic capacity. This validates increasing the photosynthetic efficiency as a much-needed strategy toward sustainably increasing the flue-cured tobacco yield, or increasing the crop yield in support of future global food security. In addition, the increases in SPAD values and *Pn* are significant for a high and stable yield of flue-cured tobacco. The results are in line with a previous report by Begum et al. [43], who reported that the tobacco yield was significantly and positively correlated with photosynthesis and SPAD values. This emphasizes the importance of optimizing photosynthetic processes and SPAD values for maximizing the tobacco yield and ensuring agricultural productivity.

Despite the positive responses of the yield to photosynthesis characteristics, some studies have reported negative responses. For example, Sinclair et al. [44] described that the photosynthesis rate does not limit crop yields in the past and is likely not to be the limiting factor for crop yields in the future, i.e., tobacco (*Nicotiana tabacum* L.) photosynthesis. However, our results are mainly attributed to biochar-based microbial fertilizer the improving soil nutrition and increasing the photosynthesis rate. This is perhaps a better way to suggest that an increased photosynthesis rate can increase the overall nitrogen uptake and storage and, thereby, contribute to increased yields [44]. This underscores the importance of considering the complex interplay between soil health, photosynthesis and nutrient availability in optimizing crop productivity.

## 5. Conclusions

Biochar-based microbial fertilizer application increased the soil water holding capacity, soil water characteristic parameters, increased gravity water (except BF_2.5_), available water and unavailable water and, in particular, available water content for the application of BF_1.5_, BF_2.5_ and BF_5.0_, which were increased by 9.47%, 1.18% and 2.19% compared to BF_0_. Excessive biochar-based microbial fertilizer application might lead to excessive aeration in soils, so it is necessary to control the ratio of applied biochar-based microbial fertilizer to prevent the excess biochar-based microbial fertilizer-induced degradation of the soil hydraulic parameters in karst mountainous areas. In addition, biochar-based microbial fertilizer improved the plant height, stem girth, leaf area, SPAD, *Pn*, *Gs*, *WUE_ins_*, *WUE_in_* and yield, while decreasing the *Ci* and *Tr* of flue-cured tobacco. However, a high ratio of BF_5.0_ had no significant effect on those traits due to the fact that it may correspond to the effective microorganisms (5%) in biochar-based microbial fertilizer-induced acidity. Thus, lower ratios (BF_1.5_ and BF_2.5_) exhibited positive influences on almost all traits, whereas a higher ratio (BF_5.0_) due to the excessive acidity had no significant influences, or the increase was not statistically significant (*p* > 0.05).

In conclusion, this study confirms that the novel biochar-based microbial fertilizer is a promising alternative to other fertilizers for tobacco cultivation, and an application of 1.5% biochar-based microbial fertilizer is recommended due to its positive influences on soil hydraulic parameters, tobacco growth/yield components and mitigation of the adverse effects of continuous cropping and excessive chemical fertilizer on plants, as well as formulating novel biochar-based microbial fertilizers that are innocuous to the karst soil environment.

## Figures and Tables

**Table 1:** Gardner fitting parameters and equation for soil water characteristic curve.

Treatment	A	B	R^2^	Fitting Equation
BF_0_	0.1761	0.1752	0.9759	*θ =* 0.1761·*ψ_m_^−^*^0.1752^
BF_1.5_	0.2001	0.1696	0.9969	*θ =* 0.2001·*ψ_m_^−^*^0.1696^
BF_2.5_	0.2262	0.1397	0.9763	*θ =* 0.2262·*ψ_m_^−^*^0.1397^
BF_5.0_	0.2170	0.1456	0.9830	*θ =* 0.1975·*ψ_m_^−^*^0.1734^

Note: *θ* is the soil water content (cm^3^ cm^−3^), *ψ_m_* is the soil matrix suction (MPa), A and B are parameters that denote the shape of the SWCC.

**Table 2:** Soil water characteristic parameters and soil water availability of different treatments.

Treatment	*θ_sat_* (cm^3^ cm^−3^)	*θ_fc_* (cm^3^ cm^−3^)	*θ_cf_* (cm^3^ cm^−3^)	*θ_pwp_* (cm^3^ cm^−3^)	*θ_hyg_* (cm^3^ cm^−3^)	*θ_gw_* (cm^3^ cm^−3^)	*θ_aw_* (cm^3^ cm^−3^)	*θ_uaw_* (cm^3^ cm^−3^)
BF_0_	0.4105	0.3500	0.2275	0.1716	0.1072	0.0605	0.1784	0.1716
BF_1.5_	0.4521	0.3869	0.2515	0.1916	0.1197	0.0652	0.1953	0.1916
BF_2.5_	0.4569	0.3989	0.2537	0.2184	0.1365	0.0580	0.1805	0.2184
BF_5.0_	0.4543	0.3923	0.2550	0.2100	0.1313	0.0620	0.1823	0.2100

Note: *θ_sat_* represent saturated moisture, *θ_fc_* represent field capacity, *θ_cf_* represent capillary fracture moisture, *θ_pwp_* represent permanent wilting point, *θ_hyg_* represent hygroscopic coefficient, *θ_gw_* represent gravity water, *θ_aw_* represent available water, *θ_uaw_* represent unavailable water.

**Table 3:** Effects of biochar-based microbial fertilizer on photosynthetic characteristics of flue-cured tobacco.

Treatment	*Pn* (μmol m^−2^ s^−1^)	*Gs* (mol m^−2^ s^−1^)	*Ci* (μmol mol^−1^)	*Tr* (mmol m^−2^ s^−1^)	*WUE_ins_* (μmol mmol^−1^)	*WUE_in_* (μmol mol^−1^)
BF_0_	9.93 ± 1.40 c	0.36 ± 0.06 a	390 ± 3.51 a	6.91 ± 0.79 a	1.48 ± 0.28 b	30.28 ± 8.43 a
BF_1.5_	15.93 ± 2.01 a	0.52 ± 0.10 a	375 ± 1.00 ab	6.89 ± 0.75 a	2.30 ± 0.05 a	36.31 ± 13.96 a
BF_2.5_	14.97 ± 1.02 ab	0.40 ± 0.04 a	367.67 ± 3.76 b	5.96 ± 0.30 a	2.51 ± 0.05 a	37.58 ± 2.43 a
BF_5.0_	10.47 ± 1.89 bc	0.39 ± 0.07 a	363.33 ± 8.29 b	3.65 ± 0.09 b	2.86 ± 0.24 a	30.31 ± 9.12 a

Note: Data are means ± S.E., *n* = 3. Different letters in the same column indicate a significant difference at the *p* = 0.05 level. BF, *Pn*, *Gs*, *Ci*, *Tr*, *WUE_ins_*, and *WUE_in_* are biochar-based microbial fertilizer, net photosynthetic rate, stomatal conductance, intercellular CO_2_ concentration, transpiration rate, leaf instantaneous water use efficiency, and leaf intrinsic water use efficiency, respectively.

**Table 4:** Pearson correlation coefficients between biochar-based microbial fertilizer and agronomic traits, SPAD, photosynthetic characteristics, WUE and yield of flue-cured tobacco.

Traits	BF	Plant Height	Stem Girth	Leaf Area	SPAD	*Pn*	*Gs*	*Ci*	*Tr*	*WUE_ins_*	*WUE_in_*	Yield
**BF**	1											
**Plant height**	0.221	1										
**Stem girth**	0.045	0.794	1									
**Leaf area**	0.044	0.983 *	0.836	1								
**SPAD**	0.122	0.982 *	0.889	0.991 **	1							
***Pn***	−0.091	0.926	0.909	0.976 *	0.975 *	1						
***Gs***	−0.062	0.871	0.479	0.879	0.813	0.791	1					
***Ci***	−0.917	−0.535	−0.438	−0.392	−0.479	−0.294	−0.167	1				
***Tr***	−0.950 *	0.092	0.231	0.269	0.194	0.396	0.322	0.757	1			
***WUE_ins_***	0.931	0.551	0.380	0.400	0.475	0.280	0.227	−0.993 **	−0.771	1		
***WUE_in_***	−0.107	0.827	0.979 *	0.891	0.918	0.963 *	0.598	−0.298	0.391	0.252	1	
**Yield**	0.101	0.984 *	0.877	0.995 **	0.999 **	0.977 *	0.831	−0.457	0.214	0.456	0.913	1

Note: * Correlation is significant at the 0.05 level; ** Correlation is significant at the 0.01 level. BF, SPAD, *Pn*, *Gs*, *Ci*, *Tr*, *WUE_ins_*, and *WUE_in_* are biochar-based microbial fertilizer, SPAD, net photosynthetic rate, stomatal conductance, intercellular CO_2_ concentration, transpiration rate, leaf instantaneous water use efficiency, and leaf intrinsic water use efficiency, respectively.

## Data Availability

Data are contained within the article.
